# Super‐resolution structure of DNA significantly differs in buccal cells of controls and Alzheimer's patients

**DOI:** 10.1002/jcp.25751

**Published:** 2017-03-28

**Authors:** Angeles Garcia, David Huang, Amanda Righolt, Christiaan Righolt, Maria Carmela Kalaw, Shubha Mathur, Elizabeth McAvoy, James Anderson, Angela Luedke, Justine Itorralba, Sabine Mai

**Affiliations:** ^1^ Department of Medicine (Geriatrics) and Neuroscience CenterQueen's UniversitySMOLKingstonOntarioCanada; ^2^ Department of Physiology and PathophysiologyManitoba Institute of Cell BiologyUniversity of ManitobaCancerCare ManitobaWinnipegManitobaCanada

**Keywords:** Alzheimer's disease, buccal cells, super‐resolution microscopy

## Abstract

The advent of super‐resolution microscopy allowed for new insights into cellular and physiological processes of normal and diseased cells. In this study, we report for the first time on the super‐resolved DNA structure of buccal cells from patients with Alzheimer's disease (AD) versus age‐ and gender‐matched healthy, non‐caregiver controls. In this super‐resolution study cohort of 74 participants, buccal cells were collected and their spatial DNA organization in the nucleus examined by 3D Structured Illumination Microscopy (3D‐SIM). Quantitation of the super‐resolution DNA structure revealed that the nuclear super‐resolution DNA structure of individuals with AD significantly differs from that of their controls (*p* < 0.05) with an overall increase in the measured DNA‐free/poor spaces. This represents a significant increase in the interchromatin compartment. We also find that the DNA structure of AD significantly differs in mild, moderate, and severe disease with respect to the DNA‐containing and DNA‐free/poor spaces. We conclude that whole genome remodeling is a feature of buccal cells in AD.

## 1 INTRODUCTION

### 1.1 Spatial organization of the nucleus

The concept of nuclear architecture linked to proper cellular function is established since the early work of Boveri (1914, 1929).

Sophisticated experimental and analytical tools have become available since then. These technological advances have enabled today's researchers to add detail to Boveri's concept. We have learned that chromosome territories are the structure of chromosomes in interphase nuclei (Cremer & Cremer, 2001; Cremer et al., 2006; Fritz et al., 2016; Sehgal et al., 2016a) and were able to depict all of them together using successive hybridizations (Bolzer et al., 2005). We learned that chromosome positions are conserved during evolution (Tanabe et al., 2002) and that gene‐poor and gene‐dense chromosomes have their specific nuclear location in human cells (Tanabe et al., 2002).

Chromosome positioning is not a constant feature of nuclei as positional changes were recorded during keratinocyte differentiation (Sehgal et al., 2016b), during adipocyte differentiation (Charó, Rodríguez Ceschan, Galigniana, Toneatto, & Piwien‐Pilipuk, 2016; Kuroda et al., 2004), after PHA‐activation of lymphocytes (Ioannou, Kandukuri, Simpson, & Tempest, 2015), and after serum deprivation/stimulation (Mehta, Amira, Harvey, & Bridger, 2010). It thus appears that the nuclear organization of the genome is directly associated with its cellular function. The first study that was able to directly link chromosome territories and their positions to cellular function was by Solovei et al. (2009). The authors demonstrated that the organization of chromosome territories differs in the rod nuclei of retinas in diurnal and nocturnal animals: In diurnal animals across the phylogenetic tree, most heterochromatin is observed at the nuclear periphery and euchromatin in the nuclear interior (Solovei et al., 2009). The rod nuclei of nocturnal animals; however, showed an inverted pattern with heterochromatin found in the nuclear center and euchromatin at the nuclear border. The authors concluded that this inverted organization of chromatin in rod cell nuclei acts as collecting lenses in nocturnal animals.

### 1.2 Super‐resolution microscopy

The 2014 Nobel Prize in Chemistry was awarded to Drs. Eric Betzig, William E. Moerner, and Stefan Hell for “the development of super‐resolved fluorescence microscopy,” a transformative breakthrough that allows imaging of objects far smaller than the Abbe diffraction‐limited spatial resolution (200 nm) and enables the study of individual molecules at ≤20 nm. While others have used this new approach to study open questions in immunology (Hu, Cang, & Lillemeier, 2016), cell biology and DNA repair (Lopez Perez et al., 2016), nuclear envelope and nuclear pores (Schermelleh et al., 2008; Xie, Horn, & Wright, 2016), in cells undergoing ischemic stress (Kirmes et al., 2015), and plant biology (Urban, Barclay, Sivaguru, Punyasena, 2016), we were the first to apply this technology to the study of the cancer cell genome (Righolt et al., 2014; Righolt, Knecht, & Mai, 2016; Sathitruangsak et al., 2015). We examined nuclear DNA organization of normal, premalignant, and malignant cells. Nuclei of cancer cells showed more DNA‐free and DNA‐poor spaces compared to normal cells (Righolt et al., 2014, 2016; Sathitruangsak et al., 2015). We developed a program to quantify the organization of nuclear DNA, using a measurement called granulometry, and applied it to the acquired super resolution images (Righolt et al., 2014, 2016; Sathitruangsak et al., 2015). In these images, the granulometry of the image (“light granulometry”) quantifies the DNA structure, and granulometry of the negative (“dark granulometry”) quantifies the spaces void of DNA structure (Righolt et al., 2014). An increase in dark granulometry shows that DNA‐poor space is increased in frequency, size, or both (Righolt et al., 2014).

### 1.3 Alzheimer's and nuclear structural markers of Alzheimer's disease

Alzheimer's disease (AD) is an increasing challenge for society. According to the Alzheimer's Society, an estimated 5.4 million Americans of all ages live with AD in 2016. This number includes an estimated 5.2 million people age 65 and older (Alzheimer's Association, 2016; Hebert, Weuve, Scherr, & Evans, 2013), and approximately 200,000 individuals under the age of 65 who have younger‐onset AD (Alzheimer's Association, 2016; Hebert et al., 2013). World‐wide, nearly 44 million people suffer from AD and related dementias (http://www.alzheimers.net/resources/alzheimers-statistics/). The costs to the health care systems are immense: Currently, the costs amount to $236 billion in the United States. In 2050, it is expected to reach $1 trillion (http://www.alz.org/facts/). No treatment for the disease that slows or reverses it has been identified to date.

In a previous study, we have focused on the spatial organization of telomeres in buccal cells of AD (Mathur et al., 2014). Buccal cells are of neuroectodermal origin and therefore considered a suitable model for studies into changes observed in the brain (Forero et al., 2016; Mathur et al., 2014; Thomas, O'Callaghan, & Fenech, 2008). Our previous study (Mathur et al., 2014) was a blinded study with 41 AD patients and 41 age‐ and gender‐matched caregivers. We measured the 3D nuclear telomere organization using TeloView (Vermolen et al., 2005) and showed that the 3D telomeric organization in buccal cells of the 82 study participants was significantly different in AD and non‐AD. Moreover, the 3D telomeric profiles were significantly different between mild, moderate, and severe AD patients. The 3D profiles described alteration in the number of telomeric signals detected, the telomere length measured and the aggregation of telomeric signals (Mathur et al., 2014). A recent meta analysis that focuses on telomere length alone has confirmed our findings of shorter telomeres in AD as a general feature of AD (Forero et al., 2016).

In the current study, we have investigated the nuclear organization of DNA in AD and non‐AD using quantitative 3D‐SIM super resolution microscopy. Our non‐AD control participants were gender‐ and age‐matched non‐caregivers as caregivers were shown to exhibit telomere shortening as a result of constant and long‐lasting daily stress (Damjanovic et al., 2007; O'Donovan et al., 2012). Our data show for the first time evidence for AD‐specific nuclear DNA organization.

## 2 MATERIALS AND METHODS

### 2.1 Study participants

Diagnosis of AD was made at the Queen's Memory Clinic according to the National Institute of Neurological and Communicative Disorders and Stroke, and the Alzheimer's Disease and Related Disorders Association (NINCDS‐ADRDA) criteria (Dubois et al., 2007; McKhann et al., 1984). Patients presented as mild, moderate, or severe AD based on their regular clinic visits and their scores on the Montreal Cognitive Assessment (MoCA) and the Mini‐Mental State Examination (MMSE) (Table 1) (Folstein, Folstein, & McHugh, 1975; McDowell, Kristjansson, Hill, & Hebert, 1997; Nasreddine et al., 1995). Patients with a MoCA score of >18/30 and/or MMSE score of ≥22/30 were categorized as mild AD. Patients with a MoCA score of ≤18/30 and/or MMSE score between 21/30 and 16/30 were considered to be in the moderate stage of AD, whereas patients with an MMSE score lower than 16/30 were classified as severe AD (Table 1) (Folstein et al., 1975; McDowell et al., 1997; Nasreddine et al., 1995). All AD patients were treated with cholinesterase inhibitors according to the Canadian guidelines for treatment of dementia (Gauthier et al., 2012; Herrmann & Gauthier, 2008). Our study examined buccal cells of 37 patients diagnosed with AD who were compared to 37 gender‐ and age‐matched cognitively normal and healthy controls. We evaluated 19 mild AD patients and their 19 controls, 14 moderate AD patients and their 14 controls, and 4 severe AD patients and their 4 controls. Since the focus of our work was on mild and moderate AD, the numbers of severe AD in this study are very low. Our study was approved by the Queen's University Research Ethics Board and by the University of Manitoba Ethic's Board (HS 16926(H2013:465)), and all 74 participants provided written informed consent.

**Table 1:** Demographics and AD scores of 74 study participants

Population	Test score ranges (MoCA/30:MMSE/30)	Number of subjects	Mean age (years ± SD)	Gender (male/female)
Mild AD	>18:≥22	19	76.4 ± 9.1	6/13
Controls, mild AD	N/A	19	75.2 ± 8.6	6/13
Moderate AD	≤18:21–16	14	77.2 ± 7.9	6/8
Controls, moderate AD	N/A	14	76 ± 13.5	6/8
Severe AD	‐‐‐‐:<16	4	85 ± 2.9	1/3
Controls, severe AD	N/A	4	81.7 ± 3.5	1/3

AD, Alzheimer's disease; MoCA, Montreal Cognitive Assessment, MMSE, Mini‐Mental State Examination.

All patients were on treatment with cholinesterase inhibitors.

### 2.2 Buccal cell collection

Buccal swabs were collected from each study participant and placed onto slides. The cells were verified to be of high quality by the Queen's Memory Clinics’ personnel and collaborating physician. Briefly, using Epicentre Catch‐A11 sample collection swabs, buccal cells were collected in duplicate from each participant and smeared onto microscope VWR pre‐cleaned frosted micro slides immediately afterward. Once slides were air‐dried, they were frozen at −20°C until shipped to the University of Manitoba on dry ice.

### 2.3 Buccal cell staining for 3D‐SIM studies

Cells placed on slides were 3D‐fixed to preserve their 3D nuclear architecture using 3.7% formaldehyde in 1 × phosphate buffered saline (1 × PBS) for 20 min at room temperature (Solovei et al., 2002). Following three washes in 1 × PBS (5 min each, shaking, at room temperature), the slides were stained with 4′,6‐diamidino‐2‐phenylindole (DAPI) (1 μg/ml) for 3 min and subsequently embedded in a drop of Vectashield (Vector Laboratories, Burlington, ON, Canada). A coverslip specially suited for super‐resolution microscopy was added (No. 1 ½, Schott, Mainz, Germany), sealed with nail polish and imaged immediately.

### 2.4 Super‐resolution imaging

3D‐SIM images were recorded as described previously (Righolt et al., 2014). Briefly, all images were acquired with a Zeiss ELYRA PS1 (Zeiss, Toronto, Ontario, Canada) equipped with a Plan‐Apochromat 63×/1.40 Oil immersion objective using an Andor EM‐CCD iXon 885 camera and a 1.6× tube lens. The DAPI channel was obtained with 405 nm laser excitation, 23 μm diffraction grating and filter cube SR Cube 07. The lateral pixel size, Δ*x* and Δ*y*, was 79 nm in the recorded images and 40 nm in the reconstructed image, the step between *z*‐planes, Δ*z*, was 91 nm. The 3D‐SIM images were reconstructed with ZEN 2012 black edition (Carl Zeiss, Jena, Germany) with the standard settings except for the regularization parameter, which was set to 10^−3^, and clipping, which was turned off. Clipping the image in the reconstruction stage was not done, because it sets the background to zero (black), hides actual image information and artificially increases the perceived resolution.

### 2.5 Image processing

The image processing and measurement steps were performed in Matlab (MathWorks, Natick, MA) with the toolbox DIPimage (Luengo Hendriks et al., 1999). A central *z*‐plane was manually selected for processing (Righolt et al., 2014). The nucleus was automatically detected by isodata thresholding (Ridler & Calvard, 1978) the widefield DAPI image and filling the holes in the binary image. The greyscale DAPI images were error‐function clipped between the 10th and 90th percentile of the intensity over the detected cell (Verbeek & van Vliet, 1993). The granulometry (3D Signatures Inc., Winnipeg, MB, Canada) of the DNA structure and DNA‐free space was measured with a morphological sieve applied to the unclipped images (Luengo Hendriks et al., 2007).

### 2.6 Nuclear aspect ratio measurements

Nuclear aspect ratios of all 2,220 nuclei were manually measured as shown in Supplemental Fig. 1. Line measurements of the “major” and “minor” lengths were done through each nucleus and the differences between the two calculated.

### 2.7 Statistical analyses

The coefficient of variation (the standard deviation divided by the mean) and skewness of the intensity histogram over the detected region was computed as well. To assess the significance of the measured difference, we used two‐sided, two‐sample Kolmogorov–Smirnov (KS) tests. A linear classification line based on the Fisher linear discriminant assuming equal priors was performed for the histogram features using the PRTools toolbox for Matlab (Duin, 2007). The structure of the DNA and DNA‐poor space were compared between cases and controls by controlling for both granular size and matching using a general linear models (GLM). Abnormal/normal cell proportions were analyzed by Mantel–Haenszel stratified analysis. The nuclear aspect ratio data were analyzed using the GLM procedure.

## 3 RESULTS

### 3.1 Buccal cell analysis of Alzheimer's disease (AD) and matched controls

We examined buccal cells of 74 study participants to define the DNA super‐resolution structure of their nuclei based on 3D Structured Illumination Microscopy (3D‐SIM), a super resolution imaging method. Table 1 summarizes the participant information and their respective Alzheimer's Disease (AD) scores (mild, moderate, or severe) based on their regular clinic visits and their scores obtained on the Montreal Cognitive Assessment (MoCA) and the Mini‐Mental State Examination (MMSE) tests (Folstein et al., 1975; McDowell et al., 1997; Nasreddine et al., 1995). Scoring of the AD study participants was carried out by a clinician (AG).

### 3.2 Super‐resolution DNA structure of buccal cells of AD and non‐AD study participants

Buccal cells of all participants were imaged using 3D‐SIM and subjected to granulometry analyses (Righolt et al., 2014). Light granulometry represents DNA structure as detected by 3D‐SIM of 4′,6‐diamidino‐2‐phenylindole (DAPI)‐stained nuclei. In contrast, dark granulometry refers to spaces void of DAPI signals and thus spaces representing DNA‐free/poor spaces detected in nuclei or the interchromatin compartment.

**Figure 1:**
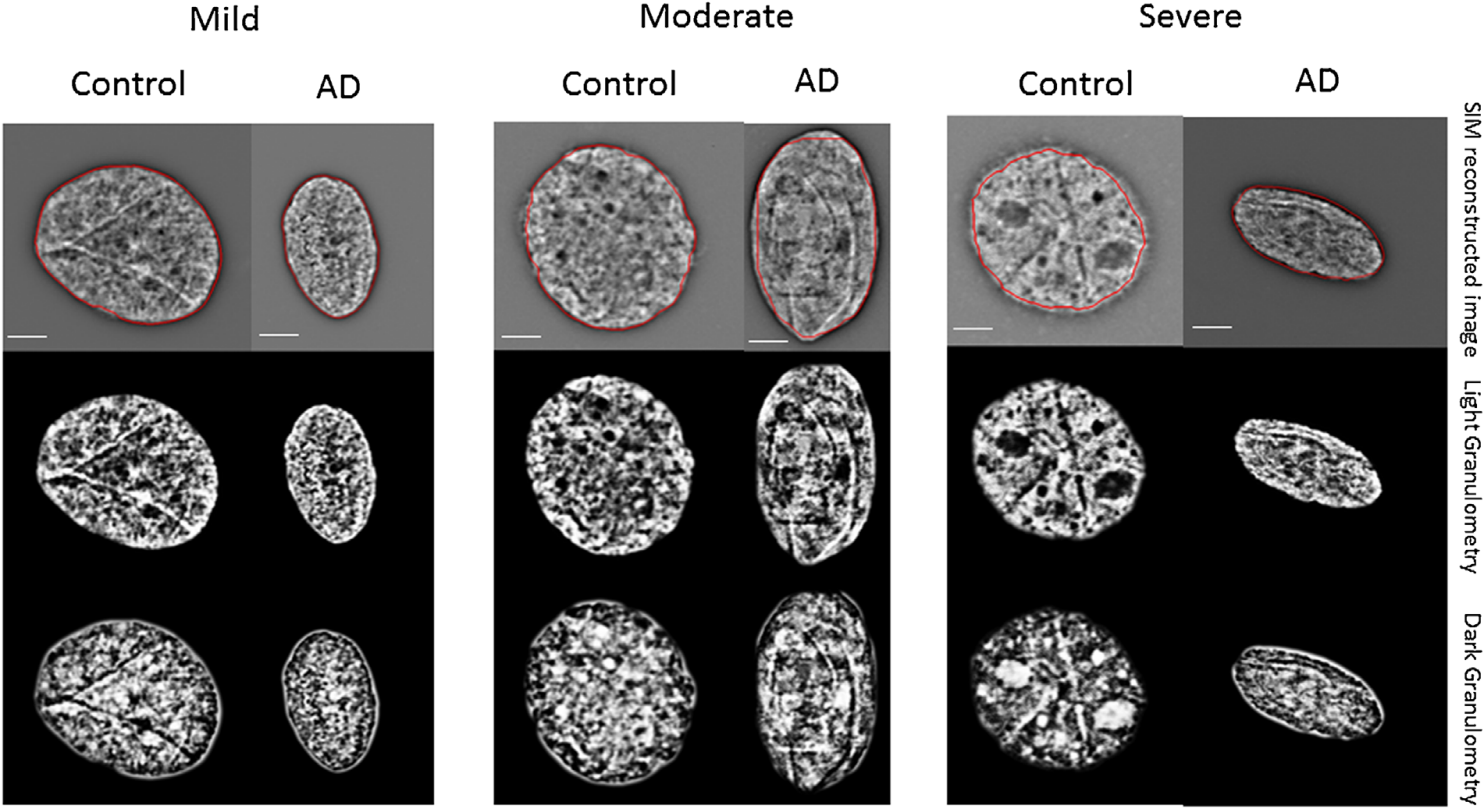
Representative 3D‐SIM images of buccal cell nuclei collected from Alzheimer's (AD) and controls and their respective light and dark granulometry images. Buccal cells were collected, placed onto slides and stained as described (Section 2). Buccal cell nuclei from mild, moderate and severe AD were compared to their age‐ and gender‐matched non‐AD controls. Top images: reconstructed 3D SIM images, middle images: light granulometry images; bottom images: dark granulometry images. For statistical analyses, see Table 2. Scale bar: 1 μm

**Figure 2:**
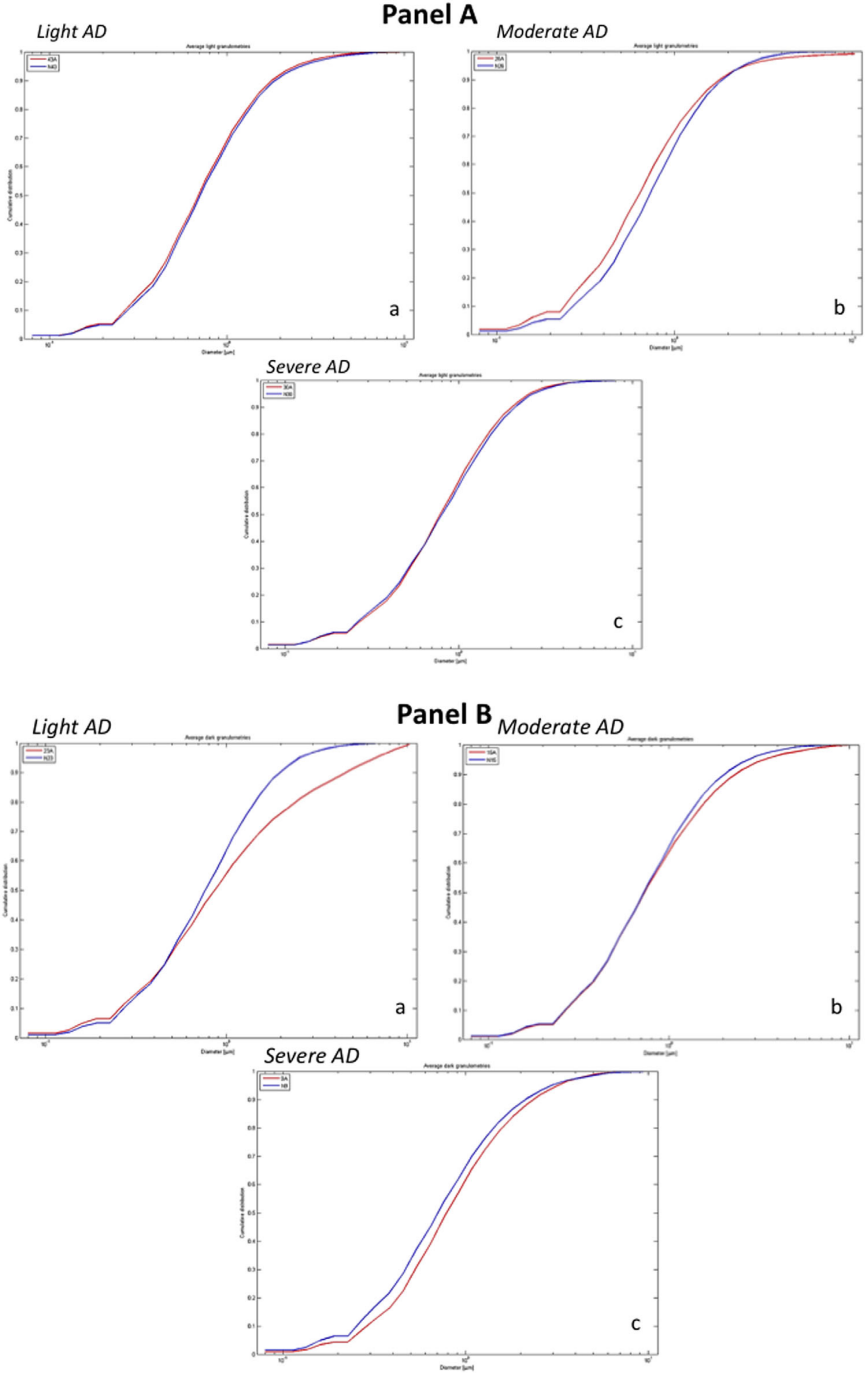
Representative light and dark granulometry measurements for buccal cell nuclei of representative individual cases of mild, moderate, and severe AD and their matched non‐AD controls. Cumulative distribution versus diameter (in μm) is given for each graph. Panel A: Light granulometries for mild (a), moderate (b), and severe AD (c) (red lines) with their respective non‐AD controls (blue lines). Panel B: dark granulometries for mild (a), moderate (b), and severe AD (c) (red lines) with their respective non‐AD controls (blue lines). The *p*‐values for the individual representative cases shown in this figure are as follows; Panel A (a): *p* = 0.75, Panel A (b): *p* = 8.98906E‐12, Panel A (c): *p* = 0.76, Panel B (a): *p* = 3.80385E‐17, Panel B (b): *p* = 0.047, Panel B (c): *p* = 7.48055E‐05. For statistical analyses of all AD versus controls in each group, see Table 2

Figure 1 illustrates the DNA structure of representative buccal cell nuclei in AD and non‐AD (dark vs. light granulometries). The organization of the DNA differs significantly between normal age‐ and gender‐matched individuals and the AD patients that scored with mild, moderate, or severe disease: Granulometry measurements were carried out (Table 2, Fig. 2). Figure 2 shows representative examples of light and dark granulometries representing the DNA‐free spaces for mild, moderate, and severe AD versus non‐AD controls, respectively (Fig. 2 Panel A and B). Statistical analyses were performed on 2,220 nuclei from 74 study participants (i.e., on 30 nuclei per person) that had been imaged with 3D‐SIM and analyzed by light and dark granulometries (Table 2). For all AD severities combined and compared to the non‐AD controls, we found significant alterations in the DNA‐poor spaces where dark granulometry increased (*p* < 0.0001). In contrast, the amount of DNA measured (light granulometry) was not significantly different (*p* = 0.6859). These data indicate that, as expected, no DNA is lost or gained between non‐AD controls and AD subjects. However, the spatial organization of the DNA in the 3D nucleus is significantly remodeled in AD leaving these nuclei with more DNA‐free/poor space and therefore a larger interchromatin compartment.

**Table 2:** Light and dark granulometry results of buccal cell nuclear DNA of 74 study participants, imaged by 3D‐SIM

	Light granulometry	Dark granulometry
All AD versus control	*p* = 0.6859	*p* < 0.0001
Mild AD versus control	*p* < 0.0001	*p* = 0.0009
Moderate AD versus control	*p* < 0.0001	*p* = 0.5704
Severe AD versus control	*p* = 0.1427	*p* < 0.0001

When individual comparisons were performed, distinctive features for each group became apparent. Buccal cells of mild AD manifest significant organizational differences in both light and dark granulometries (*p* < 0.0001 and *p* = 0.0009, respectively). Buccal cells of moderate AD display light granulometries distinct from those of non‐AD (*p* < 0.0001) but not dark granulometries (*p* = 0.5704). Buccal cells of severe AD exhibit significant differences in dark granulometries (*p* < 0.0001), while light granulometries are similar to those of the controls (*p* = 0.1427). These data indicate dynamic changes to the 3D nuclear organization of DNA during the onset and progression of the disease (Table 2).

### 3.3 Nuclear aspect ratios of buccal cells from AD and non‐AD study participants

While performing the above studies, we noted changes in the nuclear shapes of buccal cells at 3D‐SIM resolution. We examined the nuclear dimensions (minor, major, and difference) for the above 2,220 nuclei as a simple evaluation method for nuclear morphology (Section 2 and Supplemental Fig. 1). While it is known that buccal cells have several sub‐types (François et al., 2016), we have not selected for any subtype but rather collected data from all types present in a study participants’ collected sample. Combining all buccal of all AD groups compared to all buccal cells of the controls yielded significant differences in the nuclear aspect ratios (*p* = 0.0296) (Supplemental Table S1). This result indicates that the overall shape of the nuclei is more ellipsoid in buccal cells of AD than in non‐AD. When this measurement is broken down into the AD subgroups of mild, moderate, and severe, it is not a strong discriminator between the AD subgroups (Supplemental Table S1).

## 4 DISCUSSION

The data presented in this study highlight novel features of the nuclear architecture of buccal cells of patients with AD compared to gender‐ and age‐matched controls. Our data demonstrate a significant increase in DNA‐free/poor space in AD accompanied by some changes in nuclear morphology.

### 4.1 3D nuclear space and genomic remodeling in disease

This work illustrates that the 3D nuclear organization of the genome is changed in patients with AD compared to age‐ and gender‐matched controls. This finding is new for AD, but in agreement with other observations that associate the organization of the nucleus with its overall function in normal and diseased cells. While we do not know what leads to the changes in cells of individuals with AD, we have some knowledge on the genetic, environmental, and molecular mechanisms that alter the 3D nuclear organization in normal cells and in diseased cells (Charó et al., 2016; Gadji et al., 2011; Ioannou et al., 2015; Kuroda et al., 2004; Lajoie et al., 2015; Louis et al., 2005; Matarazzo, Boyle, D'Esposito, & Bickmore, 2007; Mehta et al., 2010). It is in this general context that Meaburn (2016), in a recent review, poses the question whether the spatial organization of the genome may serve as a potential biomarker of disease. She writes, “Since spatial reorganization of the genome has been identified in multiple human diseases, it is likely that spatial genome positioning patterns as a diagnostic biomarker may be applied to many diseases.”

With respect to AD, the super‐resolved organization of the genomic DNA structure may be stage‐specific as our study indicates. This conclusion complements our previous study that demonstrated the nuclear remodeling of telomeres in the 3D space of AD versus non‐AD as a stage‐specific event for mild, moderate, and severe AD versus controls (Mathur et al., 2014). In addition to the remodeling, telomeres were also significantly shorter, and this was confirmed in a recent meta‐analysis (Forero et al., 2016).

There may be dynamic changes in the organization of the genome structure and its associated function that enable the remodeling of the genome in AD. While not yet reported for AD, other studies have reported that nuclear re‐organization occurs and may be linked to neuronal differentiation and disease. In a recent review, Wilczynski (2014) summarized current evidence on large‐scale chromatin changes in neuronal differentiation, seizures, disorders of neuronal plasticity, and epilepsy. Furthermore, the role of epigenetics in AD has been studied, and several groups suggest that epigenetic changes play a role in the disease (Klein, Bennett, & De Jager, 2016; for a general review, see Alexander and Lomvardas, 2014). How epigenetic alterations exactly act upon the remodeling of DNA in the 3D space of the genome (and in AD) or how epigenetic changes drive the structural remodeling of the genome is currently unknown and warrants further investigations.

### 4.2 Super resolution DNA structure

Due to the lack of super resolved data on 3D nuclear DNA and chromosome organization, no exact knowledge about the nuclear organization of the genome existed up to very recently. Pioneering studies into the 3D spatial genome arrangement (Markaki et al., 2010) indicate that nuclei of normal cells show a network of channels and wider lacunas, called the interchromatin compartment (IC). This spatial genomic and DNA‐free network is dynamic in nature: Recent work in cells under ischemic stress has shown that the IC can expand upon ischemic stress and revert back to normal conditions (Kirmes et al., 2015; for review, see Cremer et al., 2015). Our recent work on the super resolution DNA structure in Hodgkin's lymphoma and multiple myeloma highlighted the increase in DNA‐free/poor space in nuclei of these cancer cells (Righolt et al., 2014, 2016; Sathitruangsak et al., 2015). The data of the current study on the super resolution structure of AD versus non‐AD indicate that the IC compartment increases significantly in AD. Together, all available data suggest that the previously discussed models of the interchromatin domain and interchromosomal network arrangement of the genome (reviewed in Branco & Pombo, 2006) can be tentatively answered in favor of the interchromosomal model that assumes spaces in between chromosomes. In contrast, the interchromosomal network model assumes that chromosome territories mix and form contacts with each other in the absence of interchromosomal space. This latter model is not supported by the experimental evidence revealed by super‐resolution studies. In fact, all data obtained by super resolution suggest that the IC is a dynamic feature of the genome and its overall plasticity. This holds true for AD as well as shown in the current study. In conclusion, buccal cells of AD patients show a significant increase in interchromosomal space.

## Supporting information

Additional Supporting Information may be found online in the supporting information tab for this article.

**Table S1**. Results from nuclear aspect ratio analyses of 74 participants (see Table 1).

**Figure S1**. Illustration of nuclear aspect ratio measurements.
