# Supplementary material for: Super‐resolution structure of DNA significantly differs in buccal cells of controls and Alzheimer's patients
Source: J Cell Physiol. 2017 Mar 28;232(9):2387–95. doi: 10.1002/jcp.25751 (PMC5485033; doi:10.1002/jcp.25751)
Supplement: Supplementary file 1 — Table S1. Results from nuclear aspect ratio analyses of 74 participants (see Table 1). [file JCP-232-2387-s001.docx]

**Supplemental Table 1.**

Results from nuclear aspect ratio analyses of 74 participants (see Table 1).

**Minor Major Difference**

All AD *vs*, control p=0.5717 p=0.0060 p=0.0296

Mild AD *vs*. control p=0.0022 p=0.1084 p=0.1042

Moderate AD *vs*. control p<0.0001 p<0.0001 p=0.4237

Severe AD *vs*. control p=0.2739 p=0.5302 p=0.1001
