# Supplementary figures and images for: Super‐resolution structure of DNA significantly differs in buccal cells of controls and Alzheimer's patients
Source: J Cell Physiol. 2017 Mar 28;232(9):2387–95. doi: 10.1002/jcp.25751 (PMC5485033; doi:10.1002/jcp.25751)

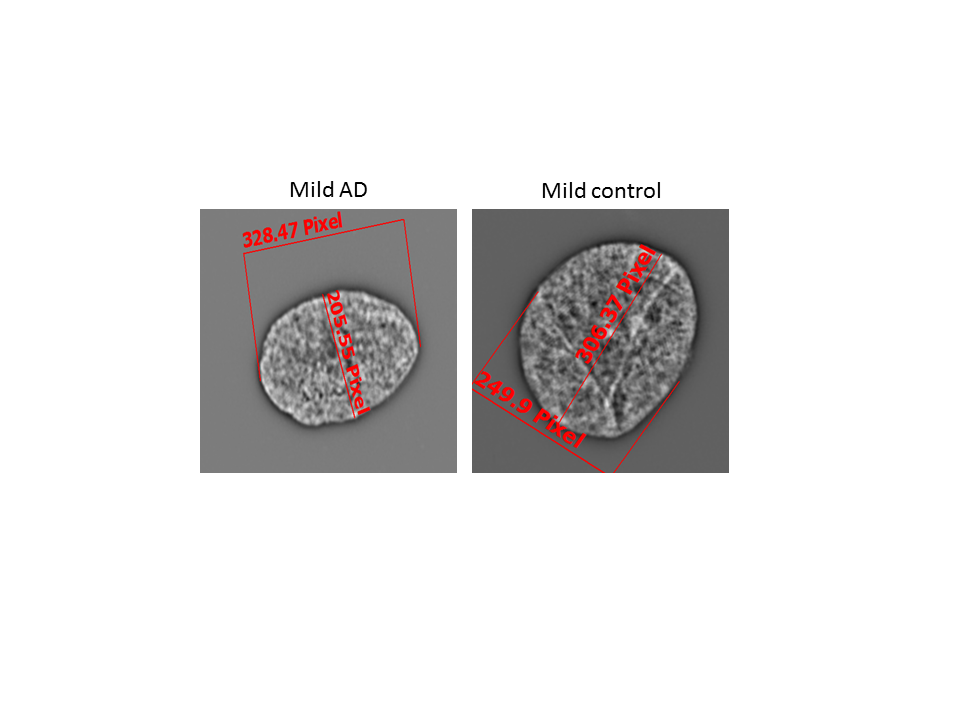

Supplement: Supplementary file 2 — Figure S1. Illustration of nuclear aspect ratio measurements. [file JCP-232-2387-s002.tif]
